# Embedded 3D Printing of Cryogel-Based Scaffolds

**DOI:** 10.1021/acsbiomaterials.3c00751

**Published:** 2023-07-18

**Authors:** Çiğdem Bilici, Mine Altunbek, Ferdows Afghah, Asena G. Tatar, Bahattin Koç

**Affiliations:** † Nanotechnology Research and Application Center, Sabanci University, Tuzla, Istanbul 34956, Turkiye; ‡ Faculty of Engineering and Natural Sciences, Sabanci University, Tuzla, Istanbul 34956, Turkiye

**Keywords:** embedded 3D printing, cryogel, GelMA, scaffold, self-recovery, alginate

## Abstract

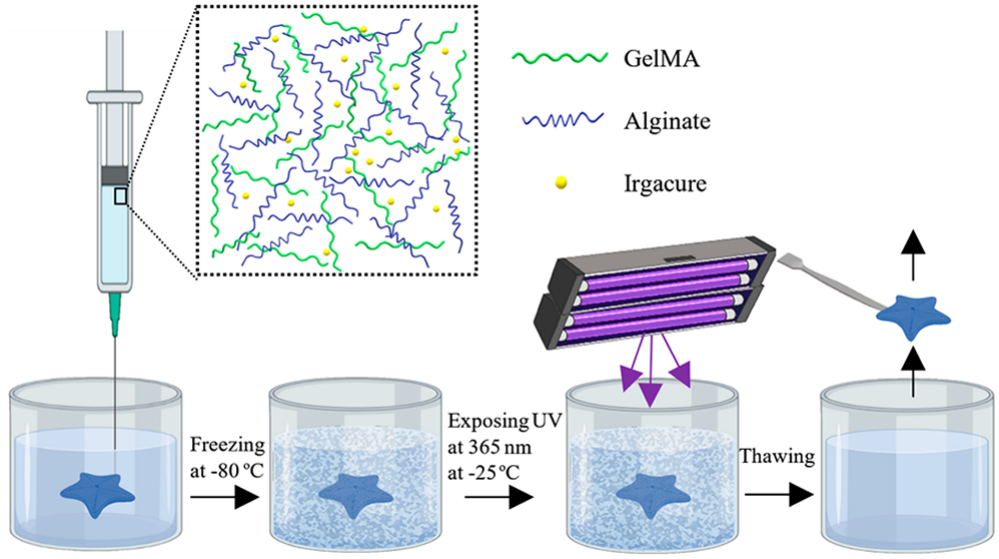

Cryogel-based scaffolds have attracted great attention in tissue engineering due to their interconnected macroporous structures. However, three-dimensional (3D) printing of cryogels with a high degree of precision and complexity is a challenge, since the synthesis of cryogels occurs under cryogenic conditions. In this study, we demonstrated the fabrication of cryogel-based scaffolds for the first time by using an embedded printing technique. A photo-cross-linkable gelatin methacryloyl (GelMA)-based ink composition, including alginate and photoinitiator, was printed into a nanoclay-based support bath. The layer-by-layer extruded ink was held in complex and overhanging structures with the help of pre-cross-linking of alginate with Ca^2+^ present in the support bath. The printed 3D structures in the support bath were frozen, and then GelMA was cross-linked at a subzero temperature under UV light. The printed and cross-linked structures were successfully recovered from the support bath with an integrated shape complexity. SEM images showed the formation of a 3D printed scaffold where porous GelMA cryogel was integrated between the cross-linked alginate hydrogels. In addition, they showed excellent shape recovery under uniaxial compression cycles of up to 80% strain. *In vitro* studies showed that the human fibroblast cells attached to the 3D printed scaffold and displayed spread morphology with a high proliferation rate. The results revealed that the embedded 3D printing technique enables the fabrication of cytocompatible cryogel based scaffolds with desired morphology and mechanical behavior using photo-cross-linkable bioink composition. The properties of the cryogels can be modified by varying the GelMA concentration, whereby various shapes of scaffolds can be fabricated to meet the specific requirements of tissue engineering applications.

## 1 Introduction

Recent studies in tissue engineering literature have mostly focused on the construction of porous 3D scaffolds to promote cell adhesion, migration, proliferation, and differentiation for repairing damaged tissue in the human body.^1−5^ Macroporous hydrogels formed at subzero temperatures, called cryogels, have great potential in tissue scaffolds, since they effectively enable cell migration and proliferation as well as oxygen and nutrient transmissions, and the removal of residual substances due to their interconnected macroporous networks.^6−8^

**Figure 1:**
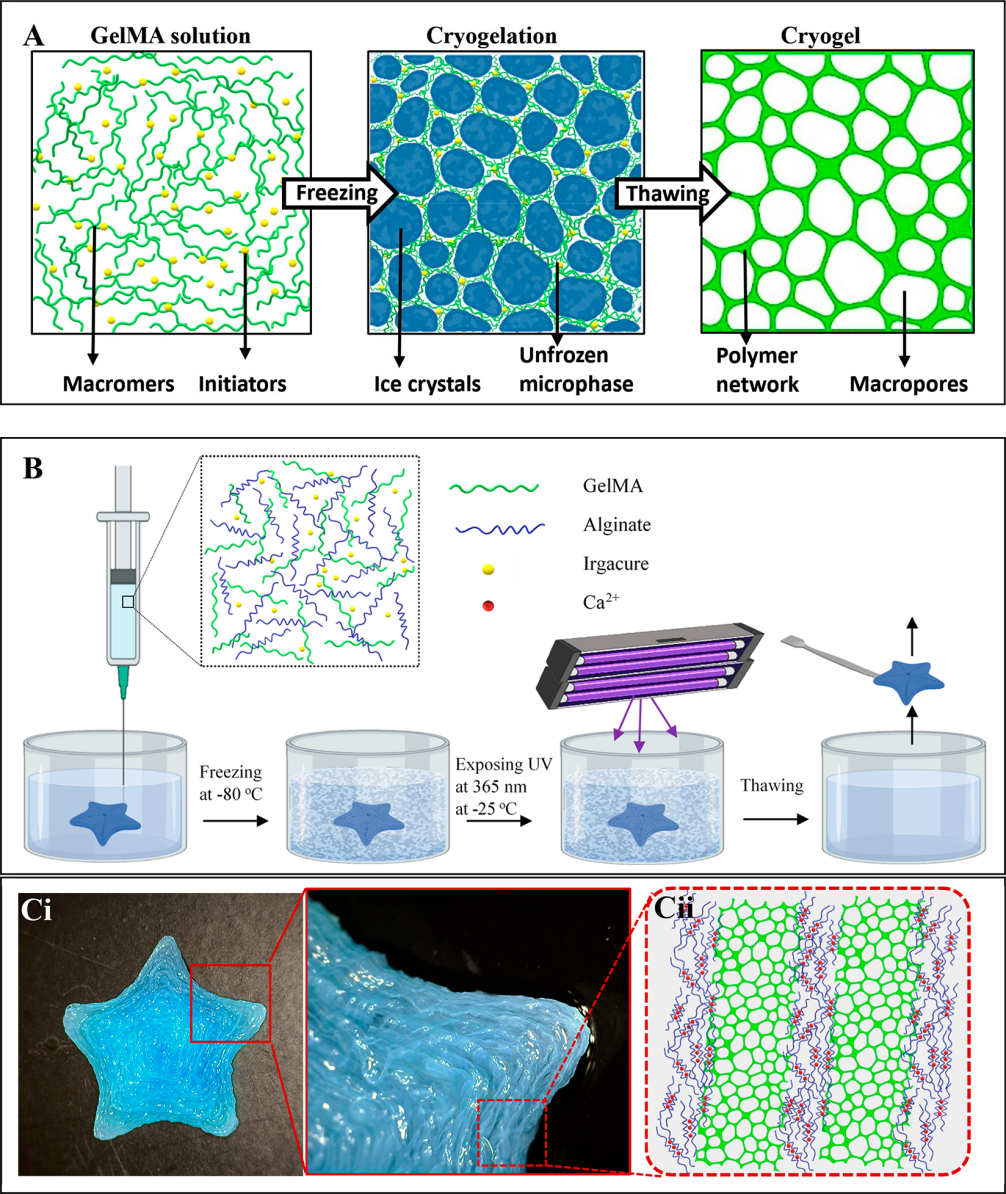
Schematic illustrations of (A) the cryogel synthesis and (B) the fabrication of cryogel-based GelMA scaffolds via embedded 3D printing. (Ci) Image of printed Starfish-shaped scaffold after recovery from support bath and (Cii) schematic representation for the hypothesized cross-linking types in the final structure illustration of the scaffold.

The biomaterial used to produce cryogel also has a significant impact on promoting cell adhesion and proliferation. Methacrylated gelatin (GelMA) is perfectly suited for producing biocompatible and biodegradable scaffolds thanks to its intrinsic cell adhesive Arg-Gly-Asp (RGD) sequence motifs and matrix metalloproteinases (MMP) recognition regions for enzymatic degradation.^9,10^ GelMA cryogels have been synthesized by cross-linking of aqueous GelMA precursor at subzero temperatures, and their use has been demonstrated for various biomedical applications including bone healing,^10−12^ regeneration of cartilage defects,^13^ drug delivery system,^14^ and vascularized pulp tissue regeneration.^15^ The schematic illustration for the basic principle of cryogel synthesis is shown in Figure 1A. During freezing, phase separation (frozen and unfrozen regions) occurs with the accumulation of GelMA macromer chains in the unfrozen region and the formation of ice crystals in the highly concentrated liquid microphase. The cross-linking reaction of GelMA macromers occurs in the unfrozen regions in the presence of initiator molecules, and the gel network is formed around large ice crystal macroparticles. Thawing ice crystals after the cross-linking of GelMA results in the formation of interconnected pores.^16^ 3D cryogel scaffolds can be obtained with different structures by performing the synthesis in shape molds, but the fabrication of complex-shaped 3D cryogels with micrometer size precision has not been achieved yet due to the mechanism of cryogelation.

3D printing technology assures higher accuracy and shape complexity for the 3D scaffolds with sophisticated geometry by fabrication of structures layer-by-layer manner.^17^ 3D cryogenic scaffolds with interconnected macropores could be fabricated by various 3D printing methods, such as the extrusion process on a cold platform^18−20^ and in a cryogenic chamber,^21^ postprinting cryogelation,^22^ and impregnation of cryogel precursor to another 3D-printed scaffold.^23^ However, 3D printing of cryogels using natural polymers, such as gelatin, alginate, collagen, silk, and hyaluronan, has hardly been performed since keeping the shape fidelity of low-viscosity natural polymer inks is challenging during extrusion without cross-linking. Embedded printing in a shear-thinning support bath could be a promising strategy to maintain the shape fidelity of low-viscosity inks which enables the deposition of complex structures.^24−26^ Through the combination of embedded printing and cryogelation, complex-shaped cryogels can be produced from low-viscosity natural polymers, such as GelMA.

In this study, for the first time, we present a 3D printing approach to fabricate macroporous complex-shaped cryogel scaffolds. The embedded 3D printing approach was combined with the cryogelation method. Photo-cross-linkable GelMA-based bioink composition were prepared by mixing varying concentrations of GelMA with alginate and Irgacure 2959, and extruded layer by layer into a Ca^2+^ containing nanoclay support bath.^27^ Stability of the printed structure was achieved by cross-linking alginate with Ca^2+^ during the extrusion, while GelMA was cross-linked under UV exposure after freezing the printed structure in the support bath. In other words, a 3D printed scaffold including hydrogel and cryogel was formed by ionic gelation of alginate at room temperature during printing and then photo-cross-linking of GelMA at −25 °C during postprinting, respectively. The 3D printed scaffolds were analyzed based on printability, integrity, geometry, mechanical properties, porosity, swelling, and degradation. In addition, *in vitro* analyses were performed to show cell attachment, migration, and proliferation to demonstrate their potential for further tissue engineering applications.

## 2 Materials and Methods

### 2.1 Materials

Gelatin (type A, 300 bloom, porcine skin), methacrylic anhydride (MA), sodium alginate (algae (marine), calcium chloride (CaCl_2_), Pluronic F127, 2-Hydroxy-4′-(2-hydroxyethoxy)-2-methylpropiophenone (Irgacure 2959), and paraformaldehyde were purchased from Sigma-Aldrich. Laponite RDS (Rheology additive based on synthetic phyllosilicate) was obtained from BYK Additives & Instruments. Dulbecco’s Modified Eagle Medium - high glucose (DMEM) and fetal bovine serum (FBS) were purchased from Sigma. Penicillin-streptomycin, phosphate buffered saline (PBS), Dulbecco’s phosphate buffered saline (dPBS), and collagenase type IV were purchased from Gibco. Human dermal fibroblast (HDF) cells were obtained from American Type Culture Collection (ATCC). Dialysis tubing (MWCO 12000–14000) was purchased from SERVAPOR(R). Calcein-AM, propidium iodine (PI), and PrestoBlue cell viability reagent were acquired from Invitrogen. 4′,6-Diamidino-2-phenylindole (DAPI) and phalloidin were bought from Abcam.

### 2.2 GelMA Synthesis

GelMA was synthesized through the reaction of gelatin with methacrylic anhydride (MA) as previously explained by Van Den Bulcke et al.^28^ Briefly, gelatin type A was dissolved in PBS at 50 °C to prepare a 10% (w/v) gelatin solution. After the dissolution of gelatin, MA was added into the solution dropwise in proportion to 0.6 g per one g gelatin and the solution was stirred for 3 h without air exposure to proceed methacrylation. Then, the solution was centrifuged at 3500 rpm for 3 min to remove unreacted residues and byproducts, and then the supernatant was separated from the pellet part. The supernatant was diluted with a 2-fold PBS solution at 40 °C to stop the reaction. The reaction solution was dialyzed against distilled water at 40 °C for at least 5 days. It was subsequently lyophilized and kept at −80 °C until further use.

### 2.3 Preparation of the Ink

The ink was prepared by dissolving alginate, GelMA, and Irgacure 2959 in deionized water. The alginate (3% w/v) and Irgacure 2959 (0.5% w/v) were used in a constant concentration, while GelMA concentration was varied between 2 and 6% w/v. Briefly, GelMA was first dissolved in deionized water at 40 °C and then Irgacure 2959 and alginate were added to the GelMA solution. 0.002% v/v food dye was added to the precursor solution to visualize the printed structures in the support bath. After dissolving homogeneously, the ink was transferred into the syringe and incubated for a half-hour at 25 ± 1 °C for printing.

### 2.4 Preparation of the Support Bath

The nanoclay composite support bath was prepared as previously described by Afghah et al.^27^ It consisted of Pluronic F127, Laponite RDS, and calcium chloride (CaCl_2_) at concentrations of 10, 3, and 0.5% w/v, respectively. Briefly, Pluronic F127 (20% w/v) was dissolved in CaCl_2_ solution (1% w/v) under stirring overnight at 4 °C. Laponite suspension (6% w/v) was prepared by dispersing Laponite RDS powder in deionized water under stirring for 1 h. Pluronic F127 solution with CaCl_2_ was slowly added into an equal volume of Laponite suspension at 4 °C and stirred until a homogeneous transparent mixture was obtained. The mixture was poured into a reservoir and incubated at 37 °C at least two h before printing.

### 2.5 3D Printing Platform and CAD Modeling

A 3D printing platform integrated into custom-engineered motion systems (Aerotech Inc.) was utilized for the fabrication of complex-shaped cryogel scaffolds. The platform was operated with an extrusion-based printing process controlled by pneumatic dispensing tools (Musashi Engineering Inc.). Computer-aided design (CAD) models of complex-shaped scaffolds were designed in Rhinoceros 6 (Robert McNeel &Associates, USA) software. 3D deposition paths were generated using a developed algorithm and transformed into G-codes. The codes were then uploaded to the motion control system of the 3D printer platform. Printing parameters were optimized in various combinations of feeding pressures, printing speed, and temperature to improve the printing capability and generate structures with high accuracy.

The ink was loaded into a plastic syringe of 10 mL in volume (Musashi Engineering, Japan) and extruded by using a double thread screwed plastic nozzle (Musashi Engineering, Japan) and a pneumatic dispenser unit (Musashi Engineering, Japan). The complex-shaped scaffolds were fabricated with a printing speed of 300 mm/min. Starfish- and nose-shaped scaffolds were printed with a 25G nozzle and 0.05 MPa of printing pressure, while tubular- and conic-shaped scaffolds were printed with a 23 G nozzle and 0.03 MPa of printing pressure. The printing of cylindrical scaffolds used for morphological and cellular analyses was carried out with a 23 G nozzle. In addition, the printing offsets between the layers were programmed with overlap because the layers must be close enough to hold the uncross-linked GelMA between them until the stage of GelMA cryogelation. For example, for the use of 25 and 23 G needles (inner diameters of 0.25 and 0.33 mm, respectively), the offset sizes of the codes were programmed to 0.23 and 0.31 mm, respectively.

### 2.6 Preparation of the 3D Printed Cryogels

After the printing process, the structures within the support bath were placed in the freezer and kept at −80 °C for 1 day. The frozen structures inside the bath were exposed to UV light with a wavelength of 365 nm for 3 h in the freezer at −25 °C. Thus, 3D printed scaffolds were fabricated. They were taken out of the freezer and kept at room temperature until the support bath thawed. After thawing, they were removed from the support bath and washed with deionized water.

### 2.7 Swelling and Degradation Tests

The swelling tests were performed on printed scaffold samples by immersing them in an excess amount of deionized water at 37 °C. After reaching the swelling equilibrium, the samples were weighed and subsequently dried with a lyophilization process. After recording the weight of dry samples, the swelling ratio, *Q*_w_ was calculated using the following formula:
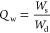
1where *W*_s_ and *W*_d_ are equilibrium swollen and dried weights of the scaffold samples, respectively.

The enzymatic degradation tests of the printed scaffolds were performed in a collagenase solution at a concentration of 0.5 mg mL^–1^. The samples of 0.1 g in weight were incubated in 2 mL of the solution on an orbital shaker with a shaking speed of 100 rpm at 37 °C. At 30 min intervals, their weights were recorded, and the weight loss was calculated by the following formula:
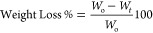
2where *W*_o_ and *W*_t_ are the weight of hydrogel samples at time 0 and time *t*, respectively.

### 2.8 Porosity Measurements

The total volume of the pores can be estimated by measuring the uptake ability of a poor solvent.^29^ Since ethanol is a poor solvent for gelatin^30^ and only occupies open pores, the freeze-dried gel samples were immersed in an excess amount of ethanol at 37 °C and the total pore volume per unit mass of the gel samples (*V*_p_) was estimated by the following formula;
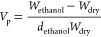
3where *W*_ethanol_, *W*_dry_, and *d*_ethanol_ are weight in ethanol, dry weight, and ethanol density (0.789 g mL^–1^), respectively.

The porosity of the scaffolds also was determined by the microcomputed tomography (μ-CT) imaging system (Bruker, SkyScan 1172, Belgium). The scaffold samples were scanned with following parameters: 50 kV voltage, 150 μA current, 180° rotation, 2 average frames, 0.20° rotation step, 20 random movement, 11.5 μm pixel resolution, 70 ms integration time, and no filter. The tomographic images were reconstructed into cross sections with NRecon software (Bruker, SkyScan,Belgium). The cross-sectional images were subsequently processed in CT-Analyzer version 1.18.4.0 (CTAn, Bruker, SkyScan, Belgium).

### 2.9 SEM Measurements

The morphological structures of the printed samples were determined by a Zeiss Leo Supra 35VP scanning electron microscope (SEM) using a secondary electron detector at 5 kV. For SEM measurements, the samples were first freeze-dried and then coated with Au–Pd under vacuum. SEM images were visualized at an accelerating voltage of 10 kV and a working distance of 10–15 mm. The morphological structures were analyzed from SEM images using ImageJ software.

### 2.10 Mechanical Tests

Uniaxial compression measurements were performed on swollen printed scaffolds by using a universal testing machine (Zwick Roell) equipped with a 200 N load cell. The scaffold samples printed in the cylinder form were compressed at 25 ± 2 °C at strain rates of 1 mm.min^–1^ up to 100% deformation. Young’s modulus *E* was calculated from the slope of the linear part of stress–strain curves between 5 and 10% compression. The compressive stress was given with nominal values, while the strain was shown with the deformation ratio. The printed gel specimens were also subjected to a compression cycle test to verify their self-recoverability. The cycle test with 5 successive loading–unloading steps was done by compressing to a maximum strain of 80% and then unloading to zero strain at the same rate.

### 2.11 *In Vitro* Cytocompatibility Analysis

The scaffolds were printed by using an ink with 4% GelMA composition for the cytocompatibility evaluation. HDF cells were used to investigate the cytocompatibility of the 3D printed cryogel-based scaffolds and their ability to provide a substrate on which the cells to grow and distribute on. The cells were cultured in DMEM supplemented with 1% l-glutamine, 1% penicillin-streptomycin, and 10% FBS and incubated at 37 °C in a humidified environment until reaching 85–90% confluency. The culture medium was refreshed every 3 days, and the cells were used under 15 passages to maintain their normal phenotype. The scaffolds were sterilized by washing with ethanol, followed by PBS wash to remove the remaining ethanol. Then, UV exposure was applied for 30 min. Scaffolds were air-dried and kept at 37 °C before cell seeding.

The cytotoxicity of the scaffolds was investigated by the treatment of HDF cells with the extract of the constructs according to ISO-993-12. In this regard, cells at passage number 11 were seeded into a 96-well plate at the density of 1 × 10^4^ cells/well and incubated for 24 h at 37 °C in a humidified environment at 95% air and 5% CO_2_. A 20 mg portion of the samples was placed in 1 mL of DMEM and incubated at 37 °C for 48 h at 75 rpm. The medium containing extract was tested on HDF cells for 24, 48, and 72 h at two dilution concentrations of 100% and 50% using a complete DMEM. Negative and positive controls were selected as only complete DMEM and 5% DMSO, respectively. Four replicates were used at each concentration. At the end of exposure time, the medium was removed, cells were washed with PBS and cell viability was assessed using a WST-1 tetrazolium colorimetric assay. The absorbance was measured by using an ELISA reader at 450 nm. Cell viability was calculated and normalized according to the absorbance of the negative control group. Experiments were performed in triplicates.

For the assessment of cell attachment and proliferation, circular shaped scaffolds with a diameter of 10 mm and thickness of 1 mm were prepared. These scaffolds were placed on a PDMS surface, and cells were seeded at a density of 2.5 × 10^5^ cells/scaffold in 100 μL media. After incubating overnight to ensure their attachment on the scaffolds, they were carefully transferred to a 24-well plate and 1 mL fresh medium was added into the wells. Cell attachment and viability were analyzed on day 7 by staining them with the Calcein AM/PI according to the manufacturer’s instruction as an indicator for positive cell attachment as well as an assessment of the cell penetration throughout the scaffold. Live and dead cells were monitored using a Carl Zeiss LSM710 confocal microscope.

To observe cell morphology and cellular distribution, we seeded HDF cells onto the scaffolds at a density of 5 × 10^5^ cells/scaffold in 100 μL of media on a PDMS surface and incubated overnight for their attachment on the scaffolds. Then, they were carefully transferred to a 24-well plate and 1 mL fresh medium was added into the wells. The scaffolds were then evaluated on day 3 and day 7 by staining their actin cytoskeleton (F-actin) and nuclei with phalloidin and DAPI, respectively. First, the 3D bioprinted scaffolds were incubated with 4% paraformaldehyde for 60 min to fix the cells within the bioprinted scaffolds. After washing with 1× dPBS, they were incubated with 0.1% Triton X-100 for 20 min to permeabilize the cell membranes, followed by another wash with dPBS. The samples were incubated with Alexa Fluor 546 Phalloidin for 60 min for staining the F-actin staining and washed with dPBS. Next, the scaffolds were incubated with DAPI for 15 min for staining of nuclei of the cells, and again washed with dPBS. Finally, the stained F-actin and cell nuclei were visualized using an inverted confocal microscope (Carl Zeiss LSM 710) with maximum excitation/emission wavelengths of 556/570 nm for F-actin staining and 358/461 nm for nuclei staining. Three dimensional images were obtained using z stacks with 5.00 μm intervals, and a pixel size of 2.77 μm.

The proliferation of cells on the cryogel-based scaffolds was analyzed by measuring the cellular metabolic activity. In brief, the printed structures were prepared in 5 × 5 × 1 mm^3^ dimensions, and a density of 2.5 × 10^4^ cell/scaffold was seeded as the steps described above. The metabolic activity of the cells was quantified on day 1, 3, and 7 using a PrestoBlue assay, as a fluorometric indicator of cell proliferation according to the manufacturer’s protocol. Briefly, PrestoBlue cell viability reagent was mixed with the culture media at the ratio of 1:10 (v/v). This solution was added onto the scaffolds at the specific time points and incubated at 37 °C for 3 h after removal of the media. Then, 200 μL of the supernatant was transferred into a black bottom 96-well plate and the fluorescence intensity was measured using a SpectraMax GEMINI XPS microplate reader at 544 nm excitation and 590 nm emission. Afterward, the scaffolds were rinsed with culture media, replaced with fresh media, and returned to 37 °C incubator until the next time point. The wells without any scaffolds were used as blank. The process was repeated for each time point and 5 replicates were used.

## 3 Results and Discussion

### 3.1 Fabrication of Cryogel-Based Scaffolds

Despite their active involvement in advanced research of polymer chemistry and biomedical applications, cryogels have not made much progress in 3D printing technology. There have been limited studies in the literature on the 3D printing of cryogels due to their preparation at subzero temperatures.^31^ In addition, because biopolymers, such as gelatin, collagen, and alginate, have a low viscosity, previously developed techniques for 3D printing of cryogels are not adequate. In this sense, we developed a unique fabrication technique for the 3D printing of cryogel scaffolds, including biopolymers.

The 3D cryogel-based scaffolds were fabricated by combining the embedded 3D printing approach with the cryogelation process. Figure 1 shows the presentation of the principle of cryogel synthesis (A) and the embedded 3D printing process for the fabrication of cryogel-based scaffold formation (B). Briefly, the GelMA-based ink including alginate and iridium chloride was deposited into the support bath containing CaCl_2_. The alginate in the ink was cross-linked with Ca^2+^ ions in the support bath during extrusion. After printing, the structure was frozen overnight at −80 °C and then exposed to UV light at 365 nm for 3 h while in the support bath at −25 °C. After cryogelation, the support bath was thawed, and the printed structure was recovered from the support bath. In this way, a printed scaffold was fabricated from the sequential cross-linking of the alginate hydrogel and GelMA cryogel. A fully integrated star-shaped scaffold was recovered from the support bath, as shown in Figure 1C. A multiphasic gel structure was observed when the scaffold was monitored in higher magnification.

Here, we hypothesized that the sequential cross-linking caused formation of a heterogeneous structure where alginate hydrogel structure was surrounded by the GelMA cryogel as schematically represented in Figure 1Cii. These multiphasic gel systems are considered to be particularly effective in modeling complicated tissue-like structures and have a critical importance for biomedical applications to enable heterogeneity, complexity, and mechanical differences.^32^ Thus, in the next section, we further characterized the structure, porosity, and mechanical strength of the cryogel-based scaffolds.

### 3.2 Macroporous Structures of the Fabricated Cryogels

**Figure 2:**
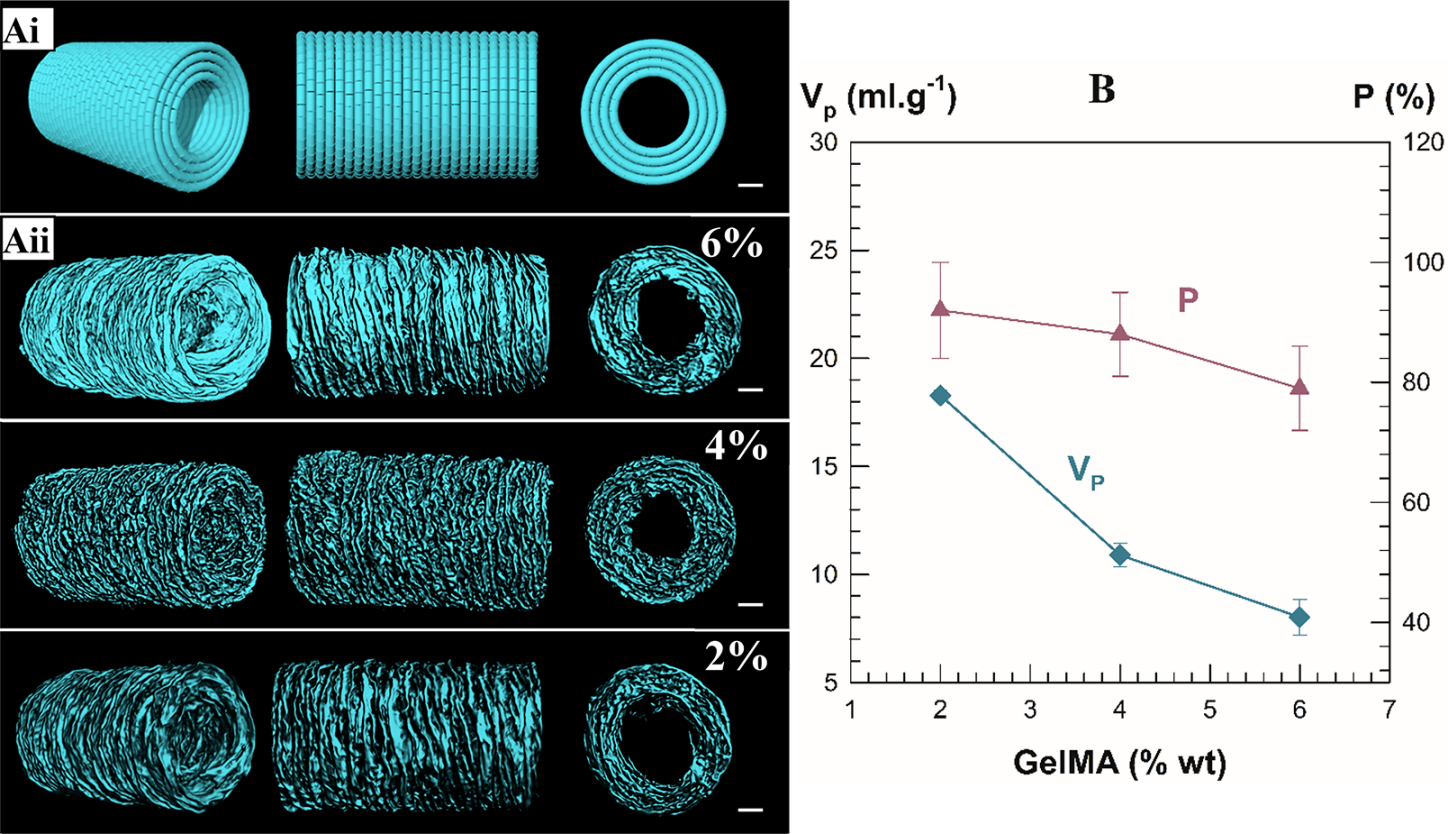
(Ai) CAD models and (Aii) μ-CT images of the hollow printed scaffolds with various GelMA concentrations. Scale bar: 1 mm. (B) The change in porosity and the total volume of the pores was based on the GelMA concentration.

The microstructure of the 3D printed cryogel-based scaffolds was characterized in terms of porosity, pore size, and interconnectivity using μ-CT. Three different ink compositions were prepared by varying the GelMA concentration to investigate the effect on the porosity of the printed scaffold. A CAD model of a hollow-shaped scaffold with 0.6 cm diameter and 1.0 cm height was used to prepare the samples for the μ-CT analysis (Figure 2Ai). The open porosity (P) of the scaffolds was measured from μ-CT analysis (Figure 2Aii). The results revealed the formation of a porous structure throughout the printing path, and P increased from 81 to 95% with decreasing GelMA concentration from 6% to 2%. It is expected that the pore size will decrease with increasing macromer concentration because an increase in GelMA concentration causes the unfrozen region where polymerization occurs to be wider than the frozen region where the pores are formed during the cryogelation process.^29,33^

The total volume of the pores (*V*_p_) was also calculated from the adsorption of poor solvent as previously described.^34,35^*V*_p_ increased from 7 to 15 mL g^–1^ as the GelMA concentration decreased from 6 to 2% w/v. The *P* and *V*_p_ were plotted as a function of GelMA concentration in Figure 2B.

**Figure 3:**
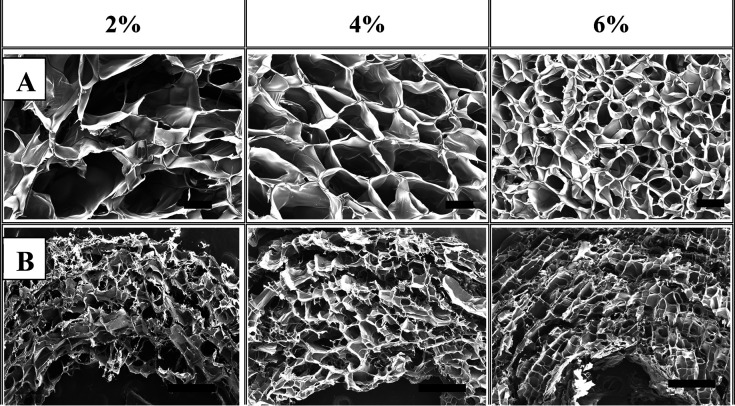
SEM images of (A) the casted cryogels and (B) 3D printed scaffolds with various GelMA concentrations. The GelMA concentration was indicated on the images. Alginate concentration was 3% (w/v) for all the printed scaffolds. Scale bars: (A) 100 μm, (B) 500 μm.

To understand the effect of GelMA concentration on pore size, the casted GelMA cryogels, which do not include alginate, were examined by SEM (Figure 3A). The average pore sizes of the casted cryogels with 2, 4, and 6% w/v GelMA were around 300, 200, and 90 μm, respectively, that is, the pore size decreased with an increase in GelMA concentration. These cryogels were described as super macroporous because their pore size is about tens and hundreds of μm, and they are attractive from the perspective of biotechnological applications.^36^ The pores of the GelMA cryogels were heterogeneously distributed, and the heterogeneity decreased with an increasing GelMA concentration. This is because the rate of gelation in the unfrozen region is faster than that of the nucleation of ice crystals in the cryogelation process when the macromer concentration is increased.^37^

The morphology of the printed scaffolds was also analyzed depending on the GelMA concentration (Figure 3B). All scaffolds were printed with a 23 G nozzle of 0.33 mm internal diameter. The cryogelation of GelMA macromer resulted in the pore formation around cross-linked alginate, which was formed in the Ca^2+^-containing support bath during the extrusion of ink. The pore size of the printed structures decreased with increasing GelMA concentration. These results indicated that the GelMA concentration plays a significant role in the porosity of the cryogel-based scaffold, while alginate hydrogel mainly supports the porous GelMA structure.

### 3.3 Characterization of 3D Printed Scaffolds

**Figure 4:**
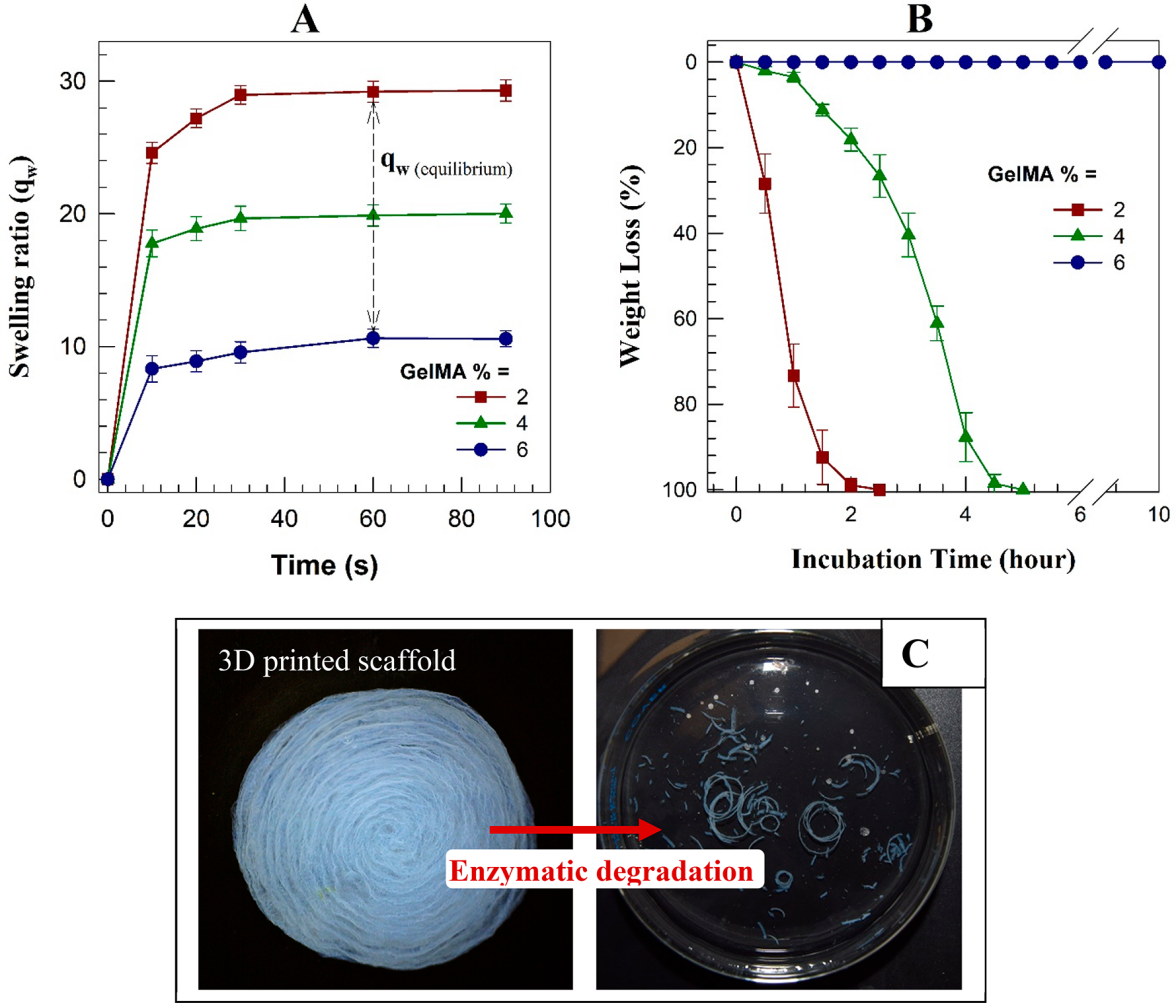
(A) Swelling ratio and (B) enzymatic degradation behavior of the printed scaffolds with various GelMA concentrations. (C) The photos of the 3D printed scaffold before (left) and after (right) enzymatic degradation with collagenase.

3D printed scaffolds were characterized by swelling, enzymatic degradation, and mechanical analyses. The swelling tests of the printed scaffolds with various GelMA concentrations were conducted by immersing them in PBS at 37 °C until they reached equilibrium swelling. The water absorption capacity of the scaffolds was evaluated with a swelling ratio by monitoring the weight loss. The swelling profiles plotted in Figure 4A showed that the swelling ratio decreased from nearly 30 to 10 with a 3-fold increase in GelMA concentration, which can be explained that due to the increase in pore size, more water could enter the pores.

In addition, the printed scaffolds reached equilibrium swelling relatively quickly compared to hydrogels, within about 1 min, because of their macroporous architectures, which enhance water diffusion into the cryogel network through the pores.

**Figure 5:**
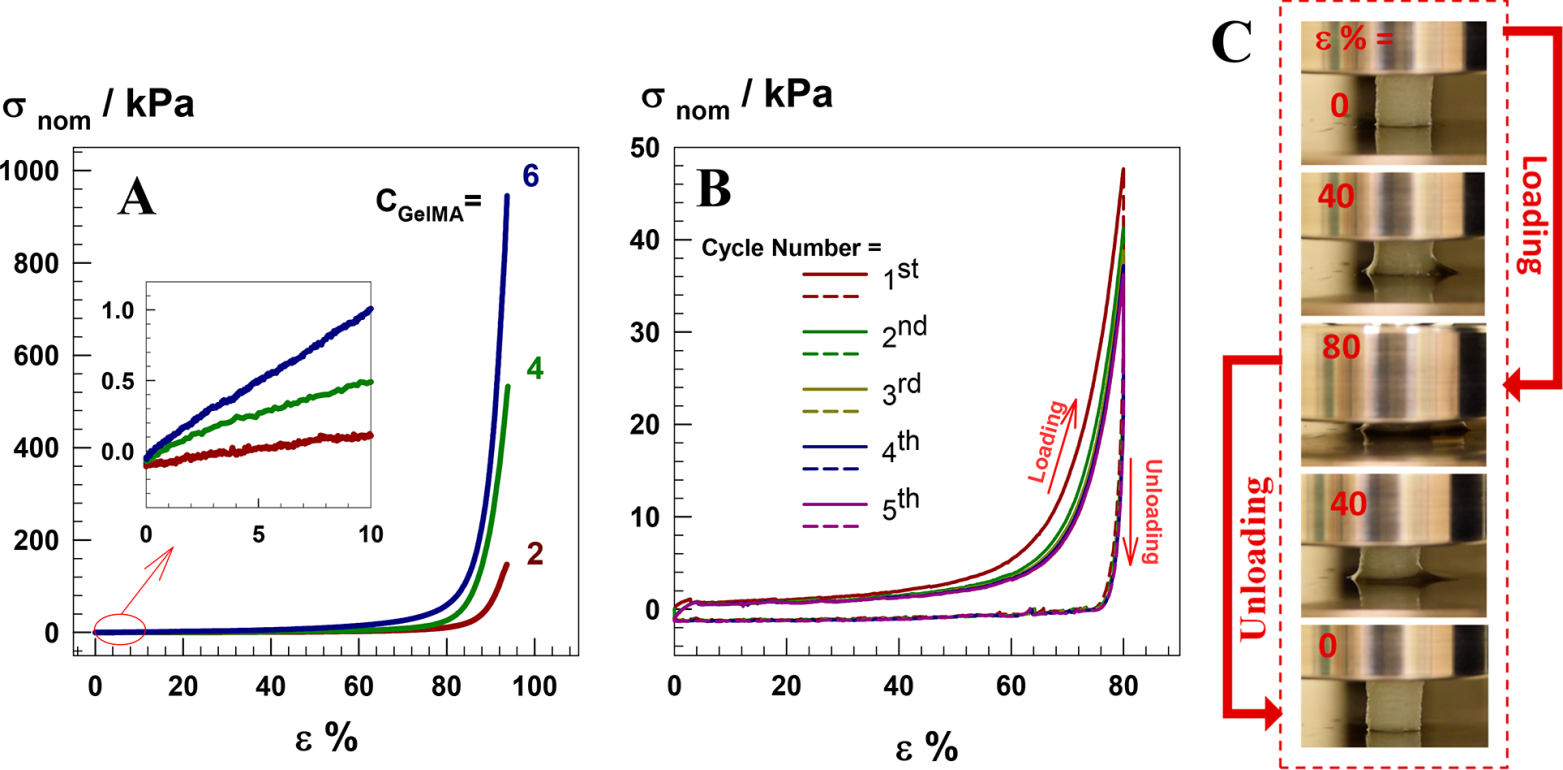
(A) Typical stress–strain curves of the printed scaffolds under compression at a strain rate of 1 mm.min^–1^. (B) Five successive loading/unloading cycles were conducted on the printed scaffold with 4% w/v GelMA. (C) The illustration of recoverability of the scaffold sample with 4% (w/v) GelMA during the loading/unloading cycle.

The printed cryogel-based scaffolds were subjected to fast enzymatic degradation for 10 h in the presence of collagenase (305 units mg^–1^) at a concentration of 0.5 mg mL^–1^. As seen in Figure 4B, the printed scaffolds with 2 and 4% GelMA degraded completely in 2.5 and 5 h, respectively, while the scaffolds with 6% GelMA resisted enzymatic degradation during the 10 h. Figure 4C represents the printed scaffold before and after enzymatic degradation. As seen in the figure, collagenase enzymes degraded only GelMA, and alginate remained like strands of a small length. These results demonstrated our hypothesis of the formation of a heterogeneous structure where alginate hydrogel strands were surrounded by the GelMA cryogel. Additionally, the higher amount of GelMA in the gel network led to a prolonged degradation process. Mechanical properties of printed scaffold samples in swollen state were investigated by uniaxial compression tests at 25 ± 2 °C. Figure 5A presents typical stress–strain curves of the compression test of the printed scaffolds prepared at various GelMA concentrations. Young’s modulus *E*, fracture stress σ_f_, and fracture strain ε_f_ of the scaffolds were measured from the stress–strain curve (Table 1). The compressive strength (0.16–0.96 MPa) and Young’s Modulus (2–13 kPa) improved with the increase in GelMA concentration in the gel network, while all scaffold samples could sustain up to about 94% compressive strain. The printed samples with 4% GelMA were also subjected to cyclic compression tests to determine their fatigue resistance against deformation (Figure 5B). The loading and unloading cycles were made 5 times up to 80% compression strain with a waiting period of 30 s between cycles. The test was performed at a constant strain rate of 1 mm min^–1^, in which the sample reabsorbs releasing water during the cycles.

**Table 1:** Mechanical Characteristics of the Printed Scaffolds Depending on GelMA Concentration

**GelMA amount (%)**	**Young’s Modulus (kPa)**	**Compressive strength (kPa)**	**Compressive strain (%)**
2	2.1 ± 0.2	150 ± 14	94.2 ± 1.5
4	4.5 ± 0.4	507 ± 44	94.2 ± 0.6
6	11.0 ± 0.8	1002 ± 105	94.0 ± 0.9

The recovery behavior of the printed scaffolds was also illustrated in Figure 5C, which was images during compressive loading up to a strain ε of 80% and unloading to zero strain. In the meantime, it was seen that the scaffold soaks the squeezed water back into its pores. The loading and unloading curves of five successive cyclic compression tests were shown in Figure 5B by the curved solid and dotted lines, respectively. The hysteresis energy dissipated (*U*_hys_) in each cycle, which indicates the recoverability of mechanical damage during loading application, was calculated from the hysteresis loop area between the loading and unloading cycles. The hysteresis energy of the first cycle is 4.2 ± 0.4 kJ/m^3^, while those of the next four cycles are about 2.7 ± 0.3 kJ/m^3^. The larger dissipation energy in the first cycle was absorbed due to irreversible fractures of the sacrificial bonds in gel network, whereas next cycles have almost the same trend as the previous ones, which stated mechanical reversibility and self-recoverability of the scaffolds.

### 3.4 Complex-Shaped Scaffolds

**Figure 6:**
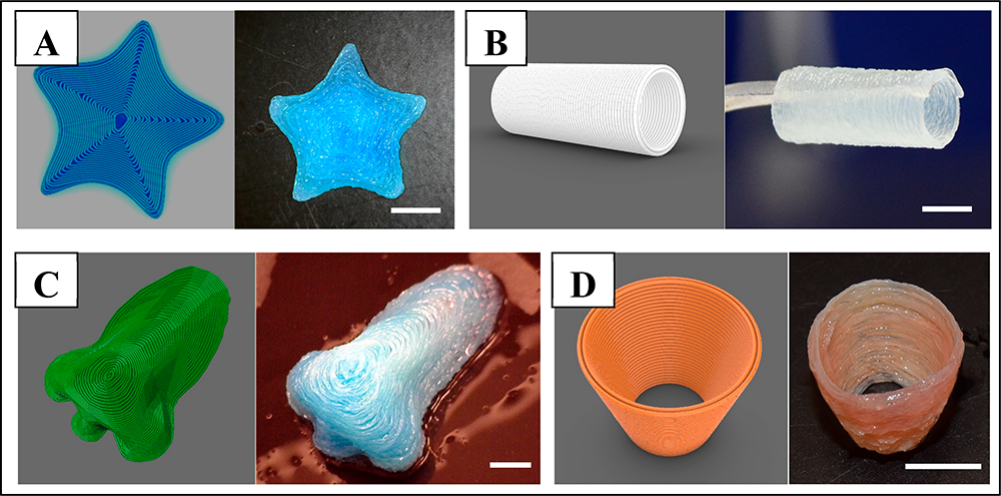
CAD models and photos of the 3D printed scaffolds including (A) starfish, (B) tubular, (C) nose, and (D) conic shapes. Scale bars: 5 mm.

The optimization of printing parameters, such as nozzle diameter, printing speed, and pressure, is a crucial step in the fabrication of the microstructural architecture of cryogel-based scaffolds with high shape fidelity. Especially the extrusion of low viscosity inks, such as GelMA ink, can result in leakage of the ink into the support bath through embedded 3D printing if these printing parameters are not adjusted accurately. Therefore, each parameter was investigated thoroughly to obtain a GelMA cryogel-based scaffold with high precision and shape fidelity. The extrusion pressure was investigated depending on each nozzle size since more pressure is required for smaller nozzle sizes, and the extrusion pressures of 0.05, 0.03, and 0.01 MPa were determined for 25, 23, and 20 G nozzles, respectively. All the printing processes were performed at a printing speed of 300 mm/min and temperatures of both ink and support bath were fixed to 25 ± 1 °C for printing. With the developed printing technique, we presented the printability of complex-shaped cryogels as macroporous scaffolds. CAD models and images of the printed various complex-shaped scaffolds, including star, tubular, conic, and nose shapes, are demonstrated in Figure 6.

In addition to the limited number of studies on 3D printing of gelatin-based cryogels, none of them have been printed in complex shapes without the use of any synthetic polymers or molds.^38−42^ Herein, we present a novel approach for 3D printing of complex-shaped GelMA cryogels, and the 3D printing approach we developed makes it possible to fabricate 3D printed biopolymer-based cryogels with complex shapes without the need to use any molds or synthetic polymer.

### 3.5 Cytocompatibility Evaluation

**Figure 7:**
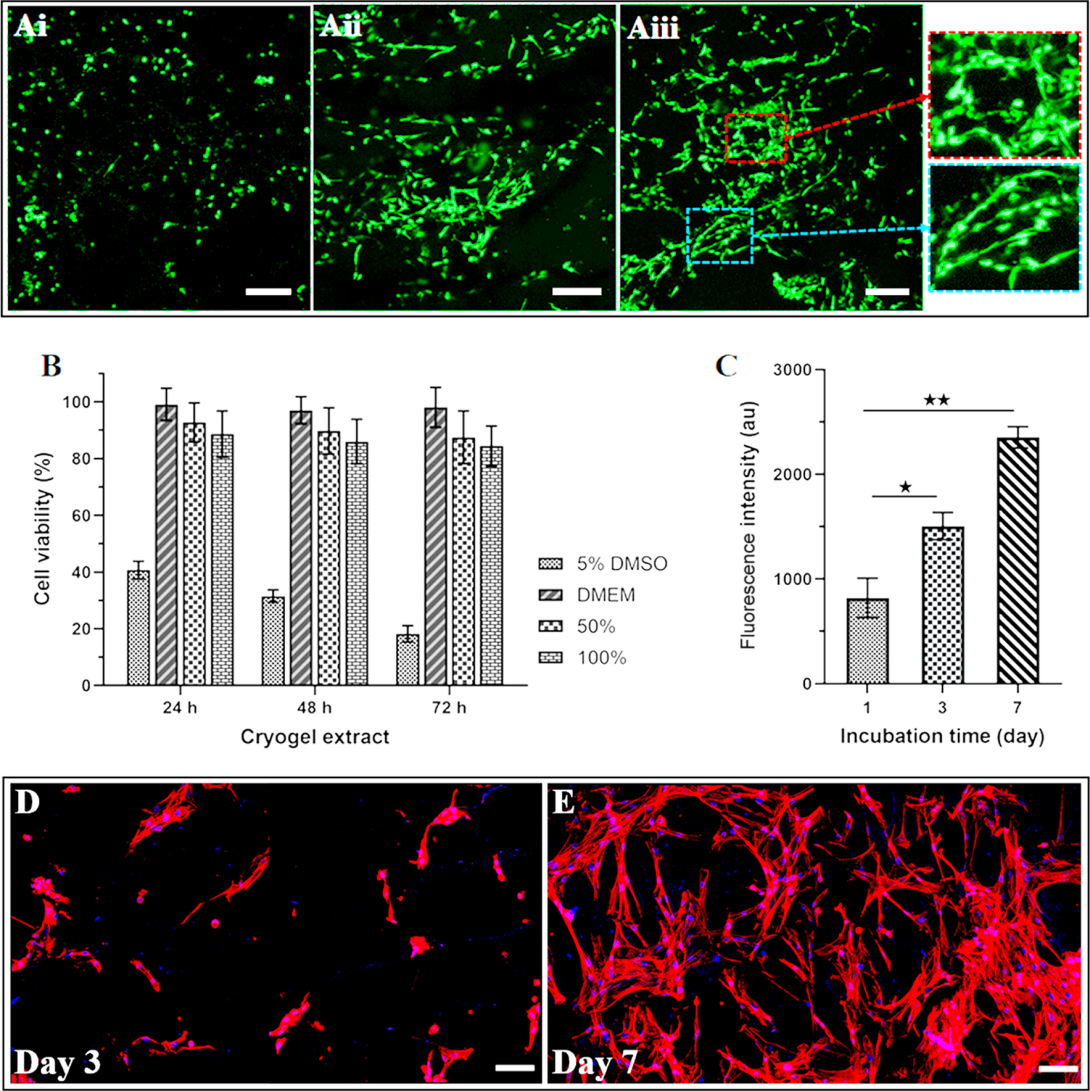
(A) Representative live/dead fluorescent image of HDF cells on the scaffold on day 1 (Ai), day 3 (Aii), and day 7 (Aiii) of incubation. Red inset: magnified view of the nonelongated cells indicating alginate portions of the structure cells. Blue inset: Zoomed view of the elongated cells representing the GelMA portion of the scaffold. (B) Cytotoxicity of the extracts of the embedded 3D printed cryogel to HDF cells. Positive control: 5% DMSO. DMEM. (C) The metabolic activity of seeded cells on the scaffolds was assessed with PrestoBlue reagent for 7 days of culture. Data reported are mean ± standard deviation with *n* = 5, ^★^*P* < 0.05, and ^★★^*P* < 0.001 were acquired using a student *t* test. Morphological analysis of cells within the scaffolds on (D) day 3 and (E) day 7 of incubation. Cell nuclei were stained with DAPI (blue), and F-actin cytoskeleton was stained with phalloidin (red). Scale bars A 200 μm, D and E 100 μm.

The biochemical and biomechanical properties and porous structure of the GelMA cryogel-based scaffolds can be useful for tissue engineering applications. They can provide a biocompatible microenvironment for cell adhesion, proliferation, and function by enabling suitable attachment sites and the transition of air and medium throughout the porous structures. We assessed i*n vitro* cytocompatibility of GelMA cryogel-based scaffolds by evaluating the attachment, viability, metabolic activity, spreading, and proliferation of HDF cells to analyze their applicability for tissue engineering applications. We selected cryogel-based scaffolds prepared with 4% GelMA and 3% alginate ink due to their moderate pore size distribution, swelling, and biodegradation properties. HDF cells were seeded onto the fabricated GelMA cryogel-based scaffolds, and their attachment to the structures and viability were envisioned for 7 days of *in vitro* culture through Calcein AM/PI staining using a confocal microscope. In Figure 7A, the attached cells were seen with green fluorescence. As shown in Figure 7A-i to 7A-ii, almost no dead cells were observed on days 1 and 3, and the number of cells rose as the incubation time increased to day 7 (Figure 7A-iii). On the other hand, HDF cells exhibited different morphologies on the different parts of the scaffold. From day 1 to day 3 of the culture, the cells started to elongate, and a few showed spindle-shaped structures. After 7 days of incubation, the cells showed spreading within the scaffolds. In some parts, they formed long elongated spindle-shape morphology while some of them stayed as a spherical shape and were not elongated on the printed structure which could be attributed to the attachment of the cells either to GelMA cryogel or alginate hydrogel parts (Figure 7A-iii) inset magnified view shown in blue dashed rectangle or red dashed rectangle, respectively).^43^ The guidance of cell alignment in a microscale transition could be promising for the construction of scaffolds for delicate tissue structures such as osteochondral tissue interfaces.^44^

Despite the cytocompatibility of the ink components including GelMA, and alginate, Irgacure 2959 used for the photo-cross-linking of the GelMA cryogel can form free-radical species during UV irradiation, which can damage the cells.^45^ Therefore, we assessed the cytocompatibility of the GelMA cryogel-based scaffolds as a critical factor. HDF cells were exposed to the extract of the structures for 72 h, and their viability was investigated using WST-1 assay. The results shown in Figure 7B, reveal that the viability of the cells is higher than 80% for all dilatations and time points. In other words, the GelMA cryogel-based scaffold has no negative effect on the cell viability.

Calcein AM staining and cytotoxicity analysis showed that HDF cells adhered and formed spindle-shaped morphologies, suggesting that the constructed structure can regulate appropriate cell behaviors, such as migration and proliferation. Thus, we investigated the proliferation of the cells in the GelMA cryogel-based scaffolds by relatively measuring the metabolic activity of cells using the PrestoBlue reagent for 7 days of incubation. The metabolic activity of the cells seeded on the printed constructs was measured quantitatively and the results show a statistically significant increase in the cellular metabolic activity from day 1 to day 3 and day 7 (Figure 7C). Metabolic activity of the cells increased more than 1.8-fold and 2.8-fold from day 1 to day 3, and from day 1 to day 7, respectively. It revealed that the cryogel-based scaffolds comprising GelMA and alginate could positively influence cell growth, subsequently leading to a higher number of cells compared to day 1. These results suggest that the structure with alginate hydrogel and porous GelMA cryogel portions provided a cytocompatible microenvironment with adequate porosity and mechanical properties in which the cells could migrate and proliferate.

The cell morphologies within the 3D printed scaffolds were assessed using Phalloidin/DAPI staining on days 3 and 7 to examine cytoskeleton organization within the 3D printed constructs. Exhibiting their appropriate cytoskeleton organization reveals that the cells can exhibit an appropriate behavior on the GelMA cryogel-based scaffold. On day 3, the cells exhibited the emergence of F-actin filaments (Figure 7D). By day 7, the samples showed clear nuclei and the formation of elongated F-actin filaments inside the cells, indicating spreading and proper organization (Figure 7E). Based on the observed cell morphologies, it is evident that this cryogel-based structure provided a favorable environment for cell proliferation and spreading. Overall, cell alignment, metabolic activity, and morphological assessment demonstrated the cytocompatibility of the proposed scaffolds for various tissue-engineering applications.

## 4 Conclusion

This study is the first to demonstrate successful embedded 3D printing of cryogel based scaffolds with proper mechanical, structural, and biological properties for tissue engineering. In addition, the 3D printing approach we developed in this study is a pioneer for the 3D printing of complex-shaped cryogel scaffolds, including biopolymers. The 3D printing of a cryogel scaffold was achieved by photo-cross-linking of frozen GelMA under UV light after printing, which were held by precross-linking of simultaneously extruded alginate with Ca^2+^ in the support bath. This sequential cross-linking led to the formation of a structure where micrometer scale hydrogel and cryogel fibers aligned sequentially in an integrated structure, hence improving the structural heterogeneity of scaffolds. Moreover, these scaffolds exhibited excellent shape recovery under compression cycles up to a strain of 80%. Depending on the GelMA concentration in the ink composition, the pore size, morphology, mechanical strength, and degradation behavior could be adjusted. The *in vitro* studies demonstrated that the cells were attached to the printed complex scaffolds, and the porous structure of the fabricated scaffold facilitated cell migration and proliferation. The developed 3D printing approach could be used for the preparation of cryogels using various low-viscosity photo-cross-linkable inks. Furthermore, by seeding HDFs on our scaffolds, we demonstrated their potential in skin tissue applications such as wound healing, skin aging, and dermal diseases.
